# Retrospective Analysis of Urinary Tract Infection in the Pediatric Population at a Tertiary Care Centre

**DOI:** 10.7759/cureus.24796

**Published:** 2022-05-07

**Authors:** Naz Perween, Sumit Rai, Sumi Nandwani, Shyam Kishor Kumar

**Affiliations:** 1 Microbiology, Post Graduate institute of Child Health, Noida, IND; 2 Microbiology, All India Institute of Medical Sciences, Mangalagiri, Mangalagiri, IND; 3 Microbiology, Post Graduate Institute of Child Health, Noida, IND; 4 Microbiology, All India Institute of Medical Sciences, Deoghar, Deoghar, IND

**Keywords:** mdr, esbl, nitrofurantoin, pyuria, uti

## Abstract

Background: Urinary tract infection (UTI) is among the most common infections occurring during childhood. It is caused by both gram-negative and gram-positive bacteria and *Escherichia coli* is the most common causative agent.

Methods: Data of all pediatric patients in the age group of 6 months to 18 years with urinary tract infection were taken for analysis. Urine samples were collected and cultured on the cystine lactose electrolyte-deficient medium. The presence of bacteria was identified using biochemicals, and the antimicrobial test was performed using the Kirby-Bauer test or the VITEK 2 compact system (bioMérieux, Inc., France).

Results: The prevalence of UTI was 23.5%. In total, 614 specimens tested positive with significant bacteriuria. The male-to-female ratio was 1:2.3. Approximately 54% patients presented with urinary symptoms alone. Culture positivity was significantly associated with pyuria (p < 0.0001). *E. coli* (334/614) was the most common isolate, followed by *Enterococcus *spp. (92/614). Colistin, polymyxin B, fosfomycin, nitrofurantoin, netilmicin, and amikacin were extremely good acting antimicrobials. Meanwhile, ampicillin, cefotaxime, ceftriaxone, and norfloxacin were highly resistant to gram-negative bacteria. Multidrug-resistant bacteria and extended-spectrum beta-lactamase-producing bacteria were found in 47% and 44.1% of cases, respectively. Vancomycin, linezolid, teicoplanin, and nitrofurantoin were highly effective against gram-positive bacteria. Furthermore, norfloxacin, trimethoprim/sulfamethoxazole, ciprofloxacin, and tetracycline were highly resistant to gram-positive bacteria. Of the 92, 42 *Enterococcus* spp. were resistant to high-dose gentamicin.

Conclusion: Nitrofurantoin and amikacin can be used as empirical therapy for gram-negative and gram-positive bacteria. Because resistance to various commonly used antibiotics is found to be increasing, treatment must be guided by antibiotic susceptibility reports.

## Introduction

Urinary tract infection (UTI) encompasses clinical conditions ranging from asymptomatic bacteriuria to severe kidney infection, which may progress to sepsis. It is one of the most common infections in the pediatric population that, if not managed appropriately, may progress to different complications such as irreversible renal parenchymal damage and renal scarring. In turn, these can cause hypertension and renal failure. The risks of UTI in childhood are approximately 1%-3% in boys and 3%-10% in girls [1]. Boys are more affected in the first few months of life than girls (2.7% vs 0.7%) [2]. However, the rate decreases thereafter. UTI is commonly characterized by burning micturition, increased urinary frequency, loss of bladder control, low back pain, and bloody or foul-smelling urine [2]. Infants cannot describe their symptoms accurately. Thus, clinicians should be more vigilant when effectively diagnosing and treating this patient group [3]. Conventional culture and sensitivity report is often delayed up to 48-72 h. Moreover, urine sample is challenging to collect in this age group, thereby making the condition more complex to manage. Hence, the treatment approach should be based on empirical therapy [4]. However, in developing countries, there is a lack of local data on antimicrobial profiles. Therefore, most clinicians treat patients according to their experience and disease severity. These random practices of antibiotic administration have posed a selection pressure on bacteria leading to the emergence of multidrug-resistant (MDR) organisms.

To date, several studies have reported about the extended-spectrum beta-lactamases (ESBLs), carbapenem-resistant *Enterobacteriaceae*, methicillin-resistant *Staphylococcus aureus* (MRSA), and vancomycin-resistant *Enterococci* (VRE) in a significant proportion of individuals [5]. The prevalence of these types of bacteria is increasing and the emergence of these superbugs has limited treatment options. Therefore, drugs should not be used based on assumptions. The epidemiological and antibiotic profiles of UTI pathogens significantly differ based on the geographical pattern. Therefore, healthcare facility personnel or treating physicians must be knowledgeable about these pathogens and local antimicrobial profiles. The current study aimed to determine the prevalence of UTI and different bacteria isolated and their antimicrobial resistance profiles, which may be helpful in determining appropriate empirical therapies.

## Materials and methods

This data-based study was performed at Post Graduate Institute of Child Health, Noida, India. All pediatric patients with complaints of UTI from the age group of 6 months to 18 years in the outpatient or inpatient department from 2017 to 2019 were included in this analysis. The requisition form received in the microbiology laboratory was used as the data collection tool to document patient details such as admission location of patients in the hospital, age, sex, symptoms, and any specific complications.

Mid-stream clean catch urine specimens were collected in a sterile container from suspected patients and were transported to the laboratory at the earliest (within two hours after collection). Proper instructions regarding sample collection were followed to prevent contamination.

Culture and identification techniques

Cystine lactose electrolyte-deficient agar (HiMedia Laboratories, Mumbai) was used to culture urine specimens with a calibrated inoculating loop with a capacity of 0.001 mL. Inoculated plates were incubated for 18-24 h at 37°C aerobically. Plates with a colony count of ≥10^5^ cfu/mL indicated significant bacteriuria [6]. Plates with three or more than three types of bacteria were considered contaminated and were not processed further. We performed further gram staining to assess the colony of interest and other biochemical tests to identify bacterial isolates.

Antimicrobial susceptibility test

Bacterial isolates underwent the antimicrobial susceptibility test (AST) using the Kirby-Bauer test or the VITEK 2 compact system (bioMérieux, Inc., France). With the disk diffusion method, three to five colonies were taken to prepare a 0.5 McFarland suspension. Then, a cotton swab was dipped into the suspension and pressed against the tube wall. It was taken out for the inoculation of the Mueller-Hinton agar plate using the lawn culture method. Plates were allowed to dry for three to five minutes, and antibiotic disks were placed on the plates using sterile forceps. These inoculated plates were incubated at 37℃ for 18-24 h. Results were interpreted according to the Clinical and Laboratory Standards Institute guidelines, 27th edition [7]. Next, the following antibiotics were tested: amikacin (30 μg), ampicillin (10 μg), ampicillin/sulbactam (10/10 μg), cefoxitin (30 μg), cefotaxime (30 μg), ceftriaxone (30 μg), ceftazidime (30 μg), cefepime (30 μg), gentamicin (10 μg), high-dose gentamicin (120 μg), imipenem (10 μg), meropenem (10 μg), nitrofurantoin (300 μg), norfloxacin (10 μg), netilmicin (30 μg), piperacillin/tazobactam (100/10 μg), trimethoprim/sulfamethoxazole (1.25/23.75 μg), aztreonam (30 μg), ciprofloxacin (5 μg), chloramphenicol (30 μg), fosfomycin (200 μg), polymyxin B (10 μg), colistin (10 μg), teicoplanin (30 μg), tetracycline (30 μg), vancomycin (30 μg), and linezolid (30 μg).

For VITEK AST, the GN72 and GP71 cards were used for non-sporing gram-negative and gram-positive bacteria, respectively. In the statistical analysis, the intermediate sensitivity results of bacterial isolates were included in the resistant results.

Quality control

*Escherichia coli* ATCC 25922, *Pseudomonas aeruginosa* ATCC 27853, *S. aureus* ATCC 25923, and *Enterococcus faecalis* ATCC 29212 strains were used for quality control.

ESBL detection

Cefotaxime (30 μg) or ceftazidime (30 μg) was used to screen ESBL-producing organisms. Isolates with a zone diameter of ≤27 mm for cefotaxime or ≤22 mm for ceftazidime were further evaluated using the double-disk diffusion method (i.e., cefotaxime 30 μg and cefotaxime/clavulanic acid 30/10 μg, or ceftazidime 30 μg and ceftazidime/clavulanic acid 30/10 μg). Lawn culture was performed on the Mueller-Hinton agar plate from the 0.5 McFarland standard of bacterial suspension. Cefotaxime (30 μg) and cefotaxime/clavulanic acid (30/10 μg) or ceftazidime (30 μg) and ceftazidime/clavulanic acid (30/10 μg) were placed on a plate placed 20 mm apart and were incubated at 37℃ for overnight. An increase in the zone diameter of ≥5 mm for either combination was considered as phenotypic confirmation for ESBL-producing organisms [7]. *E. coli* ATCC 25922 and *Klebsiella pneumoniae* 700603 were used as ESBL-negative and ESBL-positive reference strains, respectively.

Statistical analysis

Data were entered in the Microsoft Excel sheet (Microsoft, Redmond, WA). The GraphPad software (GraphPad Software, San Diego, CA) was used to analyse data. Discrete variables were expressed as percentages and proportions were compared using the Fisher’s exact test. A p value <0.05 was considered significant.

## Results

In total, 2613 specimens were collected from the patients, including 985 men and 1628 women, during a three-year period. In total, 614 specimens tested positive with significant bacteriuria (either pure or predominant growth), with a prevalence rate of 23.5%. The male-to-female ratio was 1:2.3 (p < 0.0001). Most patients came from the outpatient door (410/614, 66.8%), followed by those from the inpatient door (137/614, 22.3%) and intensive care unit (67/614, 10.9%). The preteen age group (48%) was the most commonly affected, followed by the teen age (34%), infant (11%), and preschool children (7%) groups. Approximately 54% of patients only presented with urinary symptoms such as dysuria and urgency. The other symptoms included fever without urinary symptoms (28%), fever with urinary symptoms (11%), and sepsis (7%) (Table 1).

**Table 1:** Bacterial isolates from urine samples

Bacteria	Number (percentage)
Gram-negative bacteria	
Escherichia coli	334 (54.4%)
Klebsiella pneumoniae	77 (12.5%)
Pseudomonas aeruginosa	28 (4.6%)
Proteus mirabilis	25 (4.1%)
Morganella morganii	14 (2.3%)
Alcaligenes faecalis	11 (1.8%)
Providencia stuartii	8 (1.3%)
*Citrobacter* spp.	6 (0.9%)
*Salmonella* Paratyphi A	2 (0.3%)
Serratia marcescens	2 (0.3%)
*Acinetobacter* spp.	2 (0.3%)
Pantoea agglomerans	1 (0.16%)
Enterobacter	1 (0.16%)
Burkholderia cepacia	1 (0.16%)
Gram-positive bacteria	
Enterococcus faecium	89 (14.5%)
Enterococcus faecalis	3 (0.5%)
Staphylococcus aureus	9 (1.5%)
Streptococcus	1 (0.16%)

In total, 418 patients presented with pyuria without culture positivity, and 550 patients had pyuria with culture positivity (p < 0.0001). The gram-negative bacteria (512/614) outnumbered the gram-positive bacteria (102/614). *E. coli* (334/614) was the most common isolate, followed by *Enterococcus* spp. (92/614). The other common bacteria were *K. pneumoniae *(77/614), *P. aeruginosa* (28/614), *Proteus mirabilis* (25/614), *Morganella morganii* (14/614), *Alcaligenes faecalis* (11/614), *S. aureus* (9/614), and *Providencia stuartii* (8/614) (Table 2).

**Table 2:** General characteristics of patients

Variables	Frequency	Percentage
Sex		
Boys	186	30.3%
Girls	428	69.7%
Age (years)		
<1	67	11%
1–5	43	7%
6–12	295	48%
13–18	209	34%
Location of admission		
OPD	410	66.8%
IPD	137	22.3%
ICU	67	10.9%
Symptoms		
Urinary symptoms alone	333	54%
Fever without urinary symptoms	171	28%
Fever with urinary symptoms	67	11%
Sepsis	43	7%

**Figure 1:**
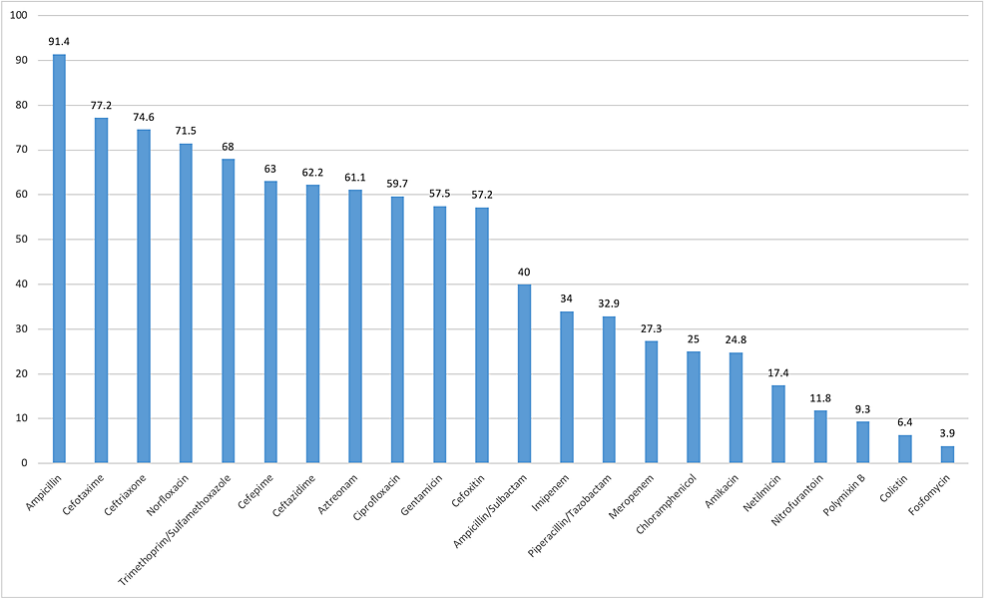
Antibiotic resistant profile of gram-negative bacteria (%)

Several antibiotics were tested against both gram-negative and gram-positive bacteria. The gram-negative bacteria were least resistant to colistin, polymyxin B, fosfomycin, nitrofurantoin, netilmicin, and amikacin and were highly resistant to ampicillin, cefotaxime, ceftriaxone, and norfloxacin (Figure 1). The four most common isolated gram-negative bacteria were showing good sensitivity for carbapenems. *P. aeruginosa* were highly resistant to commonly used cephalosporins like cefepime and ceftazidime (Table 3). A total of 240 (47%) bacteria were resistant to multiple drugs (three or more group of antibiotics). In total, 271 (44.1%) bacteria were ESBL producing, and among them, 221 (81.6%) were *E. coli*.

**Table 3:** Antibiotic resistant patterns of commonly isolated gram-negative bacteria

Antibiotics	Escherichia coli	Klebsiella pneumoniae	Proteus mirabilis	Pseudomonas aeruginosa
Ampicillin	94.4%	92.9%	63.2%	-
Cefotaxime	85.4%	63.3%	59.1%	-
Ceftriaxone	85.4%	25%	50%	-
Norfloxacin	84%	55.7%	50%	38.4%
Cefixime	80%	-	-	-
Ciprofloxacin	77.2%	33.3%	85.7%	42.3%
Trimethoprim-sulfamethoxazole	75%	55%	55.6%	-
Aztreonam	72.6%	52%	30.4%	44%
Cefepime	53.8%	100%	60%	57.7%
Ceftazidime	50%	0	66.7%	64.3%
Ampicillin-sulbactam	48.1%	52.1%	9.5%	-
Gentamicin	41.7%	0	66.7%	62.5%
Piperacillin-tazobactam	40.1%	35.4%	0	42.3%
Imipenem	39.4%	31.7%	35%	34.6%
Meropenem	30.5%	30.8%	4%	29.6%
Amikacin	24.6%	23.5%	29.2%	37%
Netilmicin	14.5%	23.8%	29.4%	38.9%
Nitrofurantoin	4.8%	24.9%	100%	50%
Fosfomycin	1.5%	3.2%	12.5%	-
Colistin	0	0	100%	7.4%
Polymyxin B	0	0	100%	5%

**Figure 2:**
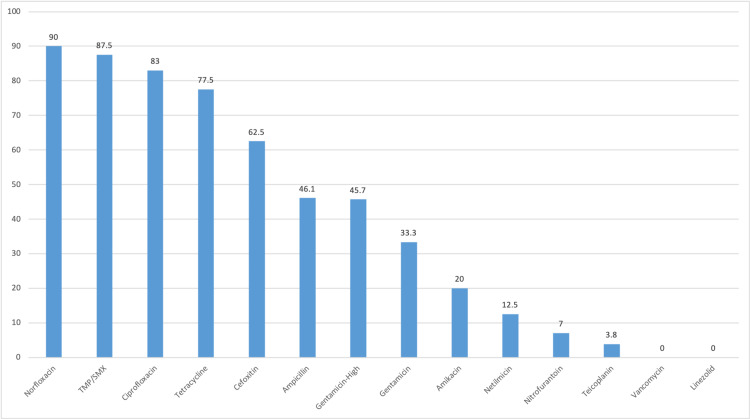
Antibiotic resistant profiles of gram-positive bacteria (%) TMP/SMX, trimethoprim/sulfamethoxazole

Vancomycin, linezolid, teicoplanin, and nitrofurantoin were least resistant to gram-positive bacteria. However, antibiotics such as norfloxacin, trimethoprim/sulfamethoxazole, ciprofloxacin, and tetracycline were highly resistant (Figure 2). Of 92, 42 *Enterococcus* spp. were resistant to high-dose gentamicin (Table 4).

**Table 4:** Antibiotic resistant patterns of commonly isolated gram-positive bacteria

Antibiotics	*Enterococcus* spp. (92)	*Staphylococcus aureus* (9)
Netilmicin	-	0
Norfloxacin	100%	100%
Ciprofloxacin	85.1%	-
Trimethoprim-sulfamethoxazole	-	100%
Cefoxitin	-	62.5%
Tetracycline	80.5%	0
Ampicillin	45.5%	-
Gentamicin	-	33.3%
High-dose gentamicin	45.1%	-
Nitrofurantoin	8.9%	0
Teicoplanin	6.5%	0
Vancomycin	1.2%	0
Amikacin	-	22.2%
Linezolid	0	0

## Discussion

This study found that the prevalence rate of UTI in the pediatric population was 23.5%. Different studies have reported prevalence rates as low as 7.8% in Iran [8] and 9% in the USA [9] and as high as 16% in Nepal [10], 26.45% in Ethiopia [2], and 21.2% and 26.7% in India [1,11]. Thus, the prevalence in our study was correlated with that of developing countries. In our study, women were more significantly affected than men, and this finding was observed in other studies as well [2,12]. The close proximity of the urethra to the anus is the major predisposing factor, which is responsible for the frequent contamination of urethral passage by fecal flora. Hence, the pathogens can easily access the bladder and kidney [2]. The preteen age groups were commonly affected in our study, and this result was in contrast to that of other studies, which revealed that toddlers are most frequently affected [13]. Most patients presented with complaints including urinary symptoms. This is in contrast to the study by Badhan et al. in which majority of the population experienced fever with urinary symptoms [11].

In 76% of cases, pyuria was observed (p < 0.0001), and this result is in contrast to that of the study by Tryphena et al. that revealed that pyuria was only observed in 22.5% of cases [1]. This microscopic finding may act as a guiding tool to start empirical therapy based on the local antibiogram, which needs to be changed after obtaining the urine culture and antimicrobial susceptibility report of patients.

Gram-negative bacteria are common intestinal habitants. Thus, they can easily colonize the perineal and periurethral area. This colonization is further facilitated by the additional structures of bacteria including flagella and pili, which are helpful for attachment with the uroepithelium and increases the risk of infection [14]. Hence, several studies showed that gram-negative bacteria are more predominant than gram-positive bacteria [2,3,14]. Furthermore, our finding is in accordance with that of other studies as we isolated 83.4% of gram-negative bacteria as pathogens. *E. coli *was the most common isolate responsible for 54.4% of infectious cases, and this finding is in accordance with that of several studies [1-3,10,14]. *Enterococcus faecium *was the second most common isolate in our study. Tryphena et al. and Shrestha et al. also isolated *Enterococcus* spp, and it was considered the second most common isolate [1,10].

In our study, gram-negative rods (GNRs) were very highly resistant to ampicillin (91.4%) and cotrimoxazole (68%), and this result was in agreement with that of other studies [1,2,15,16]. The emergence of resistance to these antibiotics might be attributed to their easy availability and frequent use as empirical therapy [2]. Our study found that GNRs were highly resistant to third- and fourth-generation cephalosporins and ciprofloxacin, which is similar to the study of Kaur et al. [16]. This might be attributed to the fact that cephalosporins and fluoroquinolones are commonly used, thereby resulting in bacteria resistance to these drugs. However, some studies have reported that cephalosporins and ciprofloxacin are sensitive drugs [2,15]. This finding may be attributed to different geographical locations and different medicinal practices. Nitrofurantoin was among the most effective antibiotics in our study. This finding is similar to that of other studies. Hence, it can be considered as empirical therapy [1,10,11,16]. Amikacin, piperacillin/tazobactam, and carbapenems also had good sensitivity, which was also observed in other studies [1,11,16]. More than 50% of GNRs were resistant to gentamicin. However, the study by Seifu and Gebissa had contrasting results, as more than 90% of GNRs were found to be sensitive to gentamicin [17]. Compared with *E. coli*, *K. pneumoniae* was more sensitive to ceftriaxone and ciprofloxacin and more resistant to nitrofurantoin in our study (Table 3).

We found that 47% of bacteria were multidrug resistant. This is alarming, and shows the inappropriate use of antibiotics in the community and healthcare facilities, lack of proper antibiotic policies, and an increased prevalence of resistant genes in bacteria. Several studies have reported a higher prevalence of MDR organisms [10,14]. In our study, ESBLs accounted for 44.1% of all organisms, which is similar to several studies [1,10,18]. However, some studies such as that conducted by Wu et al. found that only 14% of bacteria were ESBLs, and this value was significantly lower [19]. The increasing prevalence of ESBLs may be attributed to the increasing use of third-generation cephalosporin, thereby causing antibiotic selection pressure [20]. Multiple factors affect the prevalence of ESBLs such as transmissibility of ESBL strains in the community and healthcare facilities, environmental stress, and increased and inappropriate use of carbapenems, third-generation cephalosporins, and quinolones. The location of the ESBL gene on the plasmid and other mobile genetic elements facilitate the transmission of the resistant property among bacteria for multiple antibiotics, and this is another important factor [14]. The increasing percentage of ESBLs in the pediatric population limits the therapeutic options. Thus, this poses serious threat in this patient group [19].

Gram-positive bacteria were found to be highly sensitive to vancomycin, linezolid, teicoplanin, and nitrofurantoin and highly resistant to ciprofloxacin, norfloxacin, cotrimoxazole, and tetracycline in our study (Figure 2). *Enterococcus* spp. was 100% resistant to norfloxacin and highly resistant to ciprofloxacin and tetracycline (Table 4). Nitrofurantoin had a good mechanism of action against *Enterococcus* spp., and this finding is similar to that of the study conducted by Tryphena et al. However, ampicillin and high-dose gentamicin were highly resistant, which is contrary to the study results of Tryphena et al. [1]. We found VRE in 1.2% of the total *Enterococcus* spp. Different studies have shown different results. Shrestha et al. reported 5% VRE in their study while Tryphena et al. did not find any VRE [1,10]. Pouladfar et al. reported an extremely high VRE at 71.4% [21]. *S. aureus* was 100% resistant to norfloxacin and cotrimoxazole. However, it had good sensitivity to vancomycin, linezolid, teicoplanin, and amikacin (Table 4). In our study, 62.5% of *S. aureus* were methicillin resistant. Numerous studies have shown that the prevalence of MRSA in UTI is increasing [10,22]. Amikacin and gentamicin were effective against *S. aureus*.

The current study had several limitations. We did not include risk factors associated with UTI. In fact, different risk factors such as congenital anomaly or circumcision affect microbiological profiles and antibiotic resistant patterns. Moreover, we did not perform molecular tests to correlate the findings with antibiotic resistance patterns.

## Conclusions

*Escherichia coli* followed by *Enterococcus* spp. are the most common isolates of UTI. Based on this study, both gram-negative and gram-positive bacteria have a good sensitivity to nitrofurantoin and amikacin. Hence, they can be used as empirical therapy. Higher generation antibiotics such as carbapenem and colistin for gram-negative bacilli and vancomycin and linezolid for gram-positive cocci show better efficacy. However, some strains show resistant to these drugs. The prevalence of MDR and ESBL-producing bacteria is increasing. Thus, it is extremely important to use our repertoire judiciously. Furthermore, antibiotic resistance varies in different geographical locations. Hence, a local antibiogram should be followed to use for empirical therapy.
